# Local Recurrence and Breast Oncological Surgery in Young Women With Breast Cancer: The POSH Observational Cohort Study

**DOI:** 10.1097/SLA.0000000000001930

**Published:** 2016-07-27

**Authors:** Tom Maishman, Ramsey I. Cutress, Aurea Hernandez, Sue Gerty, Ellen. R. Copson, Lorraine Durcan, Diana M. Eccles

**Affiliations:** ∗ Southampton Clinical Trials Unit, University of Southampton, Southampton, United Kingdom; † Cancer Sciences Academic Unit, Faculty of Medicine, University of Southampton and University Hospital Southampton NHS Foundation Trust, Southampton, United Kingdom.

**Keywords:** breast cancer, breast conserving surgery, local recurrence, mastectomy, outcome, survival, young women

## Abstract

**Objective:**

To assess clinical and surgical factors affecting local recurrence and survival in young breast cancer patients in the Prospective study of Outcomes in Sporadic versus Hereditary breast cancer (POSH).

**Background:**

Emerging data suggest young age is a predictor of increased local recurrence.

**Methods:**

POSH is a prospective cohort of 3024 women of 18 to 40 years with breast cancer. Cohort characteristics were grouped by mastectomy or BCS. Endpoints were local-recurrence interval (LRI), distant disease-free interval (DDFI), and overall survival (OS); described using cumulative-hazard and Kaplan-Meier plots and multivariable analyses by Flexible Parametric and Cox regression models.

**Results:**

Mastectomy was performed in 1464 patients and breast-conserving surgery (BCS) in 1395. Patients undergoing mastectomy had larger tumors and higher proportions of positive family history, estrogen receptor+, progesterone receptor+, and/or human epidermal growth factor receptor 2+ tumors. Local events accounted for 15% of recurrences. LRI by surgical type varied over time with LRI similar at 18 months (1.0% vs 1.0%, *P* = 0.348) but higher for BCS at 5 and 10 years (5.3% vs 2.6%, *P* < 0.001; and 11.7% vs 4.9%, *P* < 0.001, respectively). Similar results were found in the adjusted model. Conversely, distant-metastases and deaths were lower for BCS but not after adjusting for prognostic factors. After mastectomy chest-wall radiotherapy was associated with improved LRI (hazard ratio, HR = 0.46, *P* = 0.015). Positive surgical margins, and development of local recurrence predicted for reduced DDFI (HR = 0.50, *P* < 0.001; and HR = 0.29, *P* = 0.001, respectively).

**Conclusions:**

Surgical extent appears less important for DDFI than completeness of excision or, where appropriate, chest-wall radiotherapy. Despite higher local-recurrence rates for BCS, surgical type does not influence DDFI or OS after adjusting for known prognostic factors in young breast cancer patients.

## Abstract 2

Supplemental Digital Content is available in the text

Breast cancer is the most common cancer in young adult women (age, ≤40 years) in the UK, with over 2000 new cases annually.^1^ Young women have been found to develop more aggressive tumors coupled with lower survival and higher local-recurrence rates (LRR) than older women,^2–9^ and this may be a particular issue in the developing world where a greater proportion of breast cancer appears in women of young age.^7,8^ The choice between mastectomy and breast-conserving surgery (BCS) in young women is not often a straightforward decision for clinician and/or patient.^10^ BCS is associated with better quality of life but higher LRR,^4,11^ although a meta-analysis of mostly registry and database studies in patients <40 years suggests equivalent disease-free and overall survival,^12^ whereas very young age (<35 years) has been considered a relative contraindication to BCS.^13^ Although randomized controlled trials (RCTs) suggest equivalent survival for mastectomy and BCS, very few young patients were included in these analyses.^6,14^ Indeed, young women are not routinely analyzed or reported separately in individual RCTs of BCS versus mastectomy, and any that do have very few women ≤40 years presented.^12^

Emerging evidence suggests a possible survival advantage for mastectomy in BRCA gene-mutation carriers.^15^ Young patients are more likely to be BRCA-mutation positive^5^ and retrospective cohort studies suggest that LRR are higher for BCS compared with mastectomy.^6,10,11,16^ Although family history does not affect clinical outcome in young patients, it appears to affect surgical type selection, and it is unknown if family history of breast cancer will influence local recurrence.^17^

The effect of radiotherapy plays a key role in the treatment of younger breast cancer patients. The Early Breast Cancer Trialists’ Collaborative Group (EBCTCG) found that women <40 years undergoing BCS had the highest incidence of recurrence and the largest benefit from radiotherapy; with the 10-year recurrence rate (local or distant) significantly lower compared with those without radiotherapy (36.1% vs 60.7%, respectively, *P* = 0.00009).^18^ Similar results were seen in an RCT investigating the benefit of radiotherapy boost after BCS, where the largest absolute benefit was seen in patients ≤40 years with a significant relative-risk reduction for boost (*P* = 0.003).^19^ Likewise, an RCT of premenopausal women undergoing mastectomy with/without radiotherapy showed that irradiation after mastectomy significantly improved outcomes, even after controlling for clinical and pathological factors,^20^ and a Canadian population registry study of 588 women <35 years found that LRR were reduced for postmastectomy radiation.^21^

These differences observed in the effect of radiotherapy, and the trend towards young patients having bilateral mastectomy as part of their initial cancer treatment, demonstrate that an important question remains about surgical type and outcomes in this age group. Moreover, local recurrence is very important in young breast cancer patients as there are a fewer competing risks and, other than their breast cancer, their life expectation is longer. There are no large prospective cohort studies reporting local recurrence in this age group, and a dedicated RCT comparing mastectomy versus BCS in young women is unlikely. A large prospective cohort study may offer the best level of evidence, minimizing inclusion bias, to guide management, and enable a comparison of local recurrence with disease-free survival. The Prospective study of Outcomes in Sporadic versus Hereditary breast cancer (POSH) is an observational cohort of 3000 young women with breast cancer, and is representative of the UK breast cancer population.^22^ We have not previously reported local-recurrence outcomes, and the aim of this analysis was therefore to report breast ipsilateral LRR in the POSH study to determine whether acceptable rates are found in a large cohort of young patients, and what factors, including surgical type, affect these outcomes in this age group.

## METHODS

### Study Population

POSH (MREC: 00/06/69) is a multicenter prospective observational cohort study of 3000 young women diagnosed with breast cancer in the UK between 2000 and 2008 (http://www.southampton.ac.uk/medicine/research/posh.page). All patients received treatment according to local protocols. The detailed study protocol was published in 2007,^22^ and the cohort previously described.^23^

For this analysis, type of surgery was defined as the final oncological surgery to the breast for example, if a patient had BCS followed by mastectomy ≤3 months; this was classed as a mastectomy. A mastectomy performed >3 months after primary treatment in the absence of local disease-recurrence was considered risk reducing rather than oncological. Analyses of risk-reducing surgery will be the subject of future work. Margin status was the final surgical margin after oncological operation(s), and a positive margin was defined according to American Society for Clinical Oncology (ASCO) guidance as tumor at the margin (ie, tumor on ink).^24^ This article presents analyses conducted on follow-up data from the POSH cohort received until June 26, 2015.

### Statistical Analysis

All analyses were conducted according to a prespecified plan in line with published guidance.^25^ Patients with metastatic disease at presentation were excluded. Summary statistics were used to describe the cohort and key characteristics were compared by surgical type using Pearson *χ*^*2*^ tests or Mann-Whitney *U* tests. All reported *P*-values were 2-sided.

Study endpoints were inbreast ipsilateral local-recurrence interval (LRI), distant disease-free interval (DDFI), and overall survival (OS). LRI was defined as time from date of diagnosis to date of local recurrence (either an ipsilateral recurrence or ipsilateral new primary, whichever event occurred first after BCS or chest-wall recurrence after mastectomy). The local-recurrence event was counted as an event if the date of the nonevent (death from breast cancer, distant metastases, ipsilateral local axillary recurrence, ipsilateral regional nodes recurrence, and/or contralateral recurrence, if/where applicable) was >3 months after the date of the local-recurrence event. If the date of the nonevent was ≤3 months after the local recurrence event then the patient was censored at the date of nonevent. Deaths from other cancers after local recurrence did not affect the event. DFFI was defined as time from breast cancer diagnosis to distant metastases or death from breast cancer; deaths from other causes were censored at the time of death. OS was defined as time from breast cancer diagnosis to death from any cause.

Nelson-Aalen cumulative-hazard plots were used to describe LRI and Kaplan-Meier plots were used to describe DDFI and OS. Univariable analyses (UVA) and multivariable analyses (MVA) were carried out using Cox proportional-hazards models, or Flexible Parametric Survival Models (FPSMs) for models which involved time-varying hazards.^26^ Covariates included in the MVA models included age at diagnosis (fitted as a continuous variable), tumor size, focality, nodal (N) stage, histological grade, ER and HER2 tumor status, adjuvant radiotherapy, adjuvant hormone therapy, and surgical margins, regardless of significance. Patients treated with neoadjuvant chemotherapy were included in UVA but excluded from all MVA because of difficulties in classifying pathological T and N staging for these patients. For each FPSMs, we explored varying degrees of freedom for the baseline-hazard rate and time-dependent effect to obtain the best model fit.

All analyses were performed using STATA v13.1 (StataCorp, College Station, TX, USA) on records with complete data (levels of missing data were reported).

## RESULTS

### Patient Characteristics and Definitive Surgery Information

**FIGURE 1:**
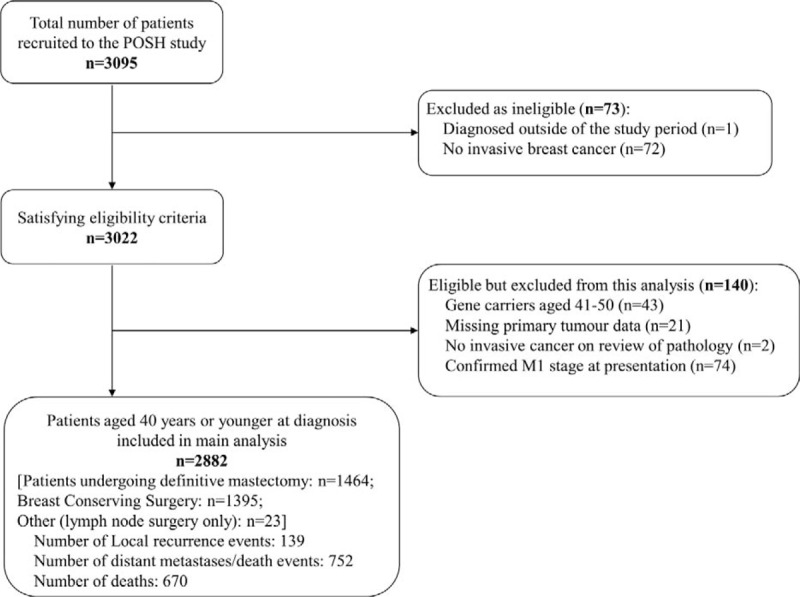
Flow chart for POSH study; local-recurrence analyses.

The POSH study recruited 3095 patients across the United Kingdom, and of 2882 included in this analysis (Fig. 1), 1464 (50.8%) underwent mastectomy and 1395 (48.4%) BCS. All patients included underwent surgery to the axilla (axillary dissection, sentinel node biopsy, or sample ± axillary dissection). Twenty-three (0.8%) patients underwent lymph node surgery only, with no surgery to the breast. Table 1 shows baseline demographics by surgical type. Median age at diagnosis was 36 years for mastectomy and BCS. Family history of breast cancer was reported significantly more for mastectomy compared with BCS (52.1% vs 48.1%, *P* = 0.037), and surveillance-detected tumors were more frequent for mastectomy than BCS (1.5% vs 0.6%). However, no significant differences were observed between surgical type for BMI and ethnicity.

**TABLE 1:** Baseline Demographic Information for All Patients by Surgery Type

Characteristic	Mastectomy	Breast Conserving Surgery	Total^*^	*P*^†^
	(n = 1464)	(n = 1395)	(n = 2882)	
Age at diagnosis, y				0.868
Median	36	36	36	
Range	18 to 40	19 to 40	18 to 40	
IQR	33 to 38	34 to 38	33 to 38	
Missing	0	0	0	
Body mass index				0.154
Median	24.5	24.8	24.6	
Range	16.5 to 59.5,	16.8 to 55.9,	16.5 to 59.5,	
IQR	22.0 to 28.1	22.1 to 28.4	22.1 to 28.4	
Missing	44 (3.0%)	64 (4.6%)	108 (3.7%)	
Race/ethnicity				0.436
White	1324 (91.9%)	1284 (93.2%)	2625 (92.4%)	
Black	63 (4.4%)	44 (3.2%)	112 (3.9%)	
Asian	43 (3.0%)	41 (3.0%)	84 (3.0%)	
Other	10 (0.7%)	9 (0.7%)	20 (0.7%)	
Missing	24 (1.6%)	17 (1.2%)	41 (1.4%)	
Family history				0.037
No	672 (47.9%)	690 (51.9%)	1378 (50.0%)	
Yes	731 (52.1%)	640 (48.1%)	1376 (50.0%)	
Missing	61 (4.2%)	65 (4.7%)	128 (4.4%)	
Presentation				<0.001
Symptomatic	1424 (97.7%)	1380 (99.4%)	2826 (98.5%)	
Screen detected	22 (1.5%)	8 (0.6%)	30 (1.0%)	
Other	12 (0.8%)	0	12 (0.4%)	
Missing	6 (0.4%)	7 (0.5%)	14 (0.5%)	
Histological grade				0.005
Grade 1	68 (4.8%)	93 (6.8%)	161 (5.7%)	
Grade 2	515 (36.2%)	429 (31.3%)	948 (33.7%)	
Grade 3	840 (59.0%)	848 (61.9%)	1703 (60.6%)	
Missing	41 (2.8%)	25 (1.8%)	70 (2.4%)	
Histological type				<0.001
Ductal	1246 (86.3%)	1230 (89.1%)	2494 (87.7%)	
Lobular	85 (5.9%)	44 (3.2%)	131 (4.6%)	
Ductal and lobular	50 (3.5%)	24 (1.7%)	74 (2.6%)	
Other	83 (5.7%)	97 (7.0%)	183 (6.4%)	
Missing	20 (1.4%)	15 (1.1%)	37 (1.3%)	
Surgical margin				<0.001
0	98 (8.9%)	113 (10.0%)	211 (9.4%)	
≥0 to <1	97 (8.8%)	126 (11.1%)	223 (10.0%)	
≥1 to ≤5	438 (39.6%)	624 (55.0%)	1062 (47.4%)	
>5	472 (42.7%)	272 (24.0%)	745 (33.2%)	
Missing	359 (24.5%)	260 (18.6%)	641 (22.2%)	
EIC^‡^				<0.001
Negative	1010 (72.8%)	1178 (86.8%)	2189 (79.7%)	
Positive	378 (27.2%)	179 (13.2%)	557 (20.3%)	
Missing	76 (5.2%)	38 (2.7%)	136 (4.7%)	
Lymphovascular invasion				<0.001
Absent	614 (45.1%)	784 (59.9%)	1402 (52.4%)	
Present	747 (54.9%)	524 (40.1%)	1276 (47.6%)	
Missing	103 (7.0%)	87 (6.2%)	204 (7.1%)	
Number of positive nodes				<0.001
0	549 (37.6%)	837 (60.5%)	1389 (48.7%)	
1–3	532 (36.5%)	404 (29.2%)	940 (33.0%)	
4–9	246 (16.9%)	99 (7.2%)	346 (12.1%)	
10+	132 (9.0%)	43 (3.1%)	175 (6.1%)	
Missing	5 (0.3%)	12 (0.9%)	32 (1.1%)	
ER status				<0.001
Negative	449 (30.7%)	516 (37.2%)	975 (34.0%)	
Positive	1013 (69.3%)	870 (62.8%)	1896 (66.0%)	
Missing	2 (0.1%)	9 (0.6%)	11 (0.4%)	
PR status				0.009
Negative	478 (40.7%)	519 (46.1%)	1008 (43.5%)	
Positive	697 (59.3%)	607 (53.9%)	1309 (56.5%)	
Missing	289 (19.7%)	269 (19.3%)	565 (19.6%)	
HER2 status				<0.001
Negative	906 (69.6%)	918 (75.9%)	1839 (72.7%)	
Positive	395 (30.4%)	292 (24.1%)	691 (27.3%)	
Missing	163 (11.1%)	185 (13.3%)	352 (12.2%)	
Focality				<0.001
Localised	707 (53.5%)	1136 (87.5%)	1845 (70.3%)	
Multifocal	615 (46.5%)	163 (12.5%)	779 (29.7%)	
Missing	142 (9.7%)	96 (6.9%)	258 (9.0%)	
Maximum invasive tumor size (mm)				<0.001
Median	28.5	19.0	22.0	
Range	0.0 to 199.0,	0.0 to 90.0,	0.0 to 199.0,	
IQR	19.0 to 43.0	14.0 to 25.0	15.0 to 33.0	
Missing	93 (6.4%)	45 (3.2%)	160 (5.6%)	
Maximum overall (invasive + in situ) tumor size, mm				<0.001
Median	37.0	20.5	27.0	
Range	0.0 to 199.0,	0.0 to 115.0,	0.0 to 199.0,	
IQR	25.0 to 55.0	15.0 to 27.0	18.0 to 40.0	
Missing	68 (4.6%)	38 (2.7%)	128 (4.4%)	
Number of operations, categorical				<0.001
1	1047 (71.5%)	1105 (79.2%)	2174 (75.4%)	
2	361 (24.7%)	279 (20.0%)	641 (22.2%)	
3	53 (3.6%)	11 (0.8%)	64 (2.2%)	
4	2 (0.1%)	0	2 (0.1%)	
5	1 (0.1%)	0	1 (0.0%)	
Missing	0	0	0	
Chemotherapy treatment period				<0.001
Adjuvant	1088 (74.3%)	1055 (75.6%)	2149 (74.6%)	
Neoadjuvant	275 (18.8%)	155 (11.1%)	447 (15.5%)	
Palliative	1 (0.1%)	0	1 (0.0%)	
Not applicable	100 (6.8%)	185 (13.3%)	285 (9.9%)	
Missing	0	0	0	
Adjuvant trastazumab				N/A
No/missing	1255 (85.7%)	1246 (89.3%)	2523 (87.5%)	
Yes	209 (14.3%)	149 (10.7%)	359 (12.5%)	
Missing	0	0	0	
Adjuvant radiotherapy				N/A
No/missing	458 (31.3%)	56 (4.0%)	525 (18.2%)	
Yes	1006 (68.7%)	1339 (96.0%)	2357 (81.8%)	
Missing	0	0	0	
Adjuvant hormone treatment				N/A
No/missing	490 (33.5%)	556 (39.9%)	1059 (36.7%)	
Yes	974 (66.5%)	839 (60.1%)	1823 (63.3%)	
Missing	0	0	0	

EIC indicates extensive intraductal component; ER, estrogen receptor; HER2, human epidermal growth factor receptor 2; IQR, interquartile range; PR, progesterone receptor; U/H, underweight/healthy.

^*^Total column includes data from the whole cohort that is, BCS, mastectomy, and other (23 patients).

^†^*P*-value obtained using Pearson *χ*^2^ test (for categorical variables) or Mann-Whitney test (for continuous variables).

^‡^EIC defined as positive where the total tumor in-situ size is ≥25% the size of the total tumor size (or where the total tumor invasive size is <75% the size of the total tumor size).

### Tumor Pathology

Significant differences in grade and focality were found between mastectomy and BCS (*P* = 0.005 and *P* < 0.001, respectively). Patients undergoing mastectomy had larger tumors were more likely to be human epidermal growth factor receptor 2+ (HER2+) and with a higher proportion of Extensive Intraductal Component positive (EIC+) compared with BCS (*P* < 0.001 in all cases).

Patients undergoing mastectomy had a significantly higher proportion of ER+ and/or PR+ tumors than BCS (estrogen receptor, ER: 69.3% vs 62.8%, *P* < 0.001; progesterone receptor, PR: 59.3% vs 53.9%, *P* = 0.009, respectively).

### Treatment and Surgery Information

Patients undergoing mastectomy had a higher frequency of negative margins compared with BCS (*P* < 0.001). Specifically, the proportion of margins >5 mm was shown to be higher (42.7% vs 24.0%, respectively), whereas the proportion of margins 1 to 5 mm was lower for BCS (39.6% vs 55.0%, respectively).

Although the median number of operations was one for mastectomy and BCS, the distribution was significantly different (*P* < 0.001), with a higher proportion of patients undergoing mastectomy having more than one surgery (28.5% vs 20.8%, *P* < 0.001). Only 11.1% of BCS patients underwent neoadjuvant chemotherapy compared with 18.8% for mastectomy. Adjuvant radiotherapy was given to 68.7% of patients undergoing a mastectomy. In 56 patients undergoing BCS, we were unable to confirm adjuvant breast radiotherapy. We cannot exclude that these patients had radiotherapy to the breast at a different center and that the information was not recorded, nor can we confirm that these patients did not receive adjuvant breast radiotherapy. However, these patients were analyzed as not having radiotherapy. Nine-hundred (61.5%) patients undergoing mastectomy had chest-wall radiotherapy (CWR-XRT) whereas 977 (70.0%) patients undergoing BCS had a radiotherapy boost. In patients undergoing BCS, no clear association was seen between margin status (>0/negative vs 0/positive) and provision of a radiotherapy boost (data not shown).

Missing data were similar across surgical types and low for most demographic information. Exceptions were PR, HER2, and surgical margin information, with up to 19.7%, 13.3%, and 24.5% missing, respectively.

### Follow Up and Survival

**FIGURE 2:**
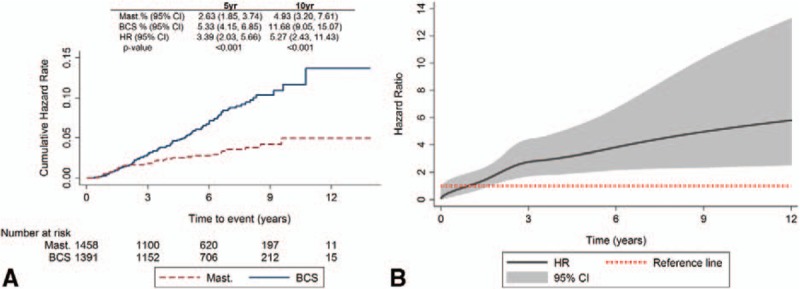
Local-recurrence interval for all patients by surgical type. A, Nelson-Aalen cumulative hazard plot. B, Flexible parametric survival model time-varying hazard over time.

Median follow up was 7.3 years for mastectomy, BCS, and overall. There were 139 local-recurrence events compared with 752 DDFI events overall, demonstrating that the majority of events experienced were because of distant metastases or death from breast cancer (Fig. 1). Ninety-five local-recurrence events were experienced for patients undergoing BCS (6.8% of these patients), compared with just 40 (2.7%) for mastectomy. Two-hundred and sixty DDFI events were experienced for BCS (18.6%), and 485 (33.1%) for mastectomy. Similar numbers were found for OS, with 232 (16.6%) and 431 (29.4%) for BCS and mastectomy, respectively. Figure 2A shows the Nelson-Aalen cumulative-hazard rates for LRI by surgical type. There was no significant difference in the estimated 18-month LRR between mastectomy and BCS (hazard ratio, HR: 1.43; 95% confidence interval, CI: 0.89–2.32; *P* = 0.143). However, patients undergoing BCS had a significantly higher LRR at 5 years (2.63% vs 5.33%; HR, 3.39; 95% CI, 2.03–5.66; *P* < 0.001) and at 10 years (4.93% vs 11.68%; HR, 5.27; 95% CI, 2.43–11.43; *P* < 0.001). The change in HR over time is illustrated in Fig. 2B, with the HR crossing one at 12 months. Similar results were found when excluding patients with a maximum overall (invasive + in situ) tumor diameter >30 mm (thus excluding patients with larger tumors who were likely to be treated with a mastectomy) (data not shown). MVA showed that patients undergoing BCS also had a significantly higher chance of a local recurrence at both 5 and 10 years, with only adjuvant radiotherapy significantly affecting the MVA (Table 2). When looking at LRI for mastectomy by CWR-XRT (Supplementary Figure 1A), patients without CWR-XRT had a significantly higher LRR compared with those with CWR-XRT (HR, 0.46; 95% CI, 0.24–0.86; *P* = 0.015). However, when assessing LRI for BCS patients by radiotherapy boost, no significant differences were found between patients with/without a boost (HR, 0.90; 95% CI, 0.58–1.38; *P* = 0.614) (Supplementary Figure 1B). There was also no difference for BCS for those with surgical margins of 0 mm versus >0 mm in terms of LRI (HR, 0.86; 95% CI, 0.41–1.78; *P* = 0.680) (Supplementary Figure 1C).

**TABLE 2:** Local Recurrence Interval Flexible Parametric Survival Model Multivariable Analysis Results for all Patients (Excluding Those With Neoadjuvant Chemotherapy)

Covariate	HR^*^	95% CI	*P*
Surgical type at 5 years			
Mastectomy	1 (Ref. cat.)	—	—
BCS (unadjusted)	5.33	4.15 to 6.85	<0.001
BCS (adjusted)	5.00	3.57 to 23.69	<0.001
Surgical type at 10 years			
Mastectomy	1 (Ref. cat.)	—	—
BCS (unadjusted)	11.68	9.05 to 15.07	<0.001
BCS (adjusted)	6.06	1.29 to 28.40	0.022
Age at diagnosis, y, (continuous)	1.02	0.95 to 1.09	0.662
Maximum overall (invasive + in situ) tumor size, (mm) (continuous)	1.42	0.78 to 2.58	0.253
Focality			
Localized	1 (Ref. cat.)	—	—
Multifocal	1.15	0.58 to 2.30	0.688
N stage			
N0	1 (Ref. cat.)	—	—
N1	1.18	0.71 to 1.96	0.527
Histological grade			
1	1 (Ref. cat.)	—	—
2	1.63	0.38 to 7.02	0.514
3	1.42	0.33 to 6.16	0.636
ER status			
Negative	1 (Ref. cat.)	—	—
Positive	0.64	0.28 to 1.48	0.297
HER2 status			
Negative	1 (Ref. cat.)	—	—
Positive	1.33	0.77 to 2.30	0.306
Adjuvant radiotherapy			
No/unknown	1 (Ref. cat.)	—	—
Yes	0.32	0.16 to 0.64	0.001
Adjuvant hormone therapy			
No/unknown	1 (Ref. cat.)	—	—
Yes	0.64	0.28 to 1.47	0.295
Surgical margins, mm			
0	1 (Ref. cat.)	—	—
≥0 to <1	0.74	0.26 to 2.14	0.579
≥1 to ≤5	0.93	0.40 to 2.18	0.871
>5	0.89	0.36 to 2.22	0.803

CI indicates confidence interval; ER, estrogen receptor; HER2, human epidermal growth factor receptor 2; HR, hazard ratio.

^*^Unless otherwise stated, HR presented for the multivariable (adjusted) model.

**FIGURE 3:**
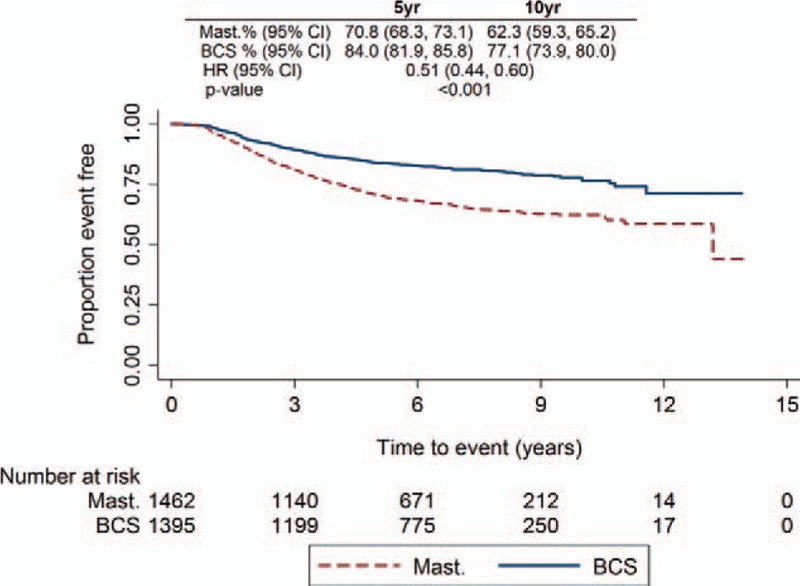
Distant disease free interval Kaplan-Meier plot for all patients by surgical type.

**FIGURE 4:**
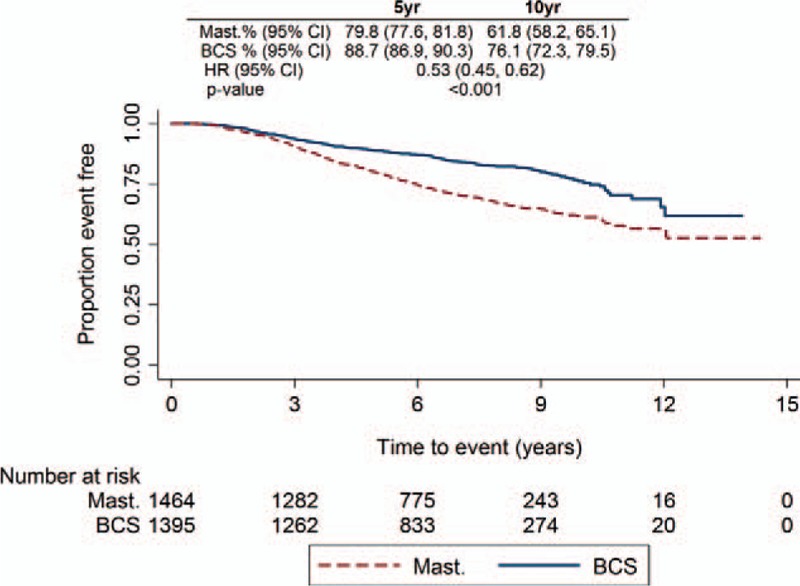
Overall survival Kaplan-Meier plot for all patients by surgical type.

DDFI by surgical type showed that mastectomy patients had a significantly worse DDFI than BCS (HR, 0.51; 95% CI, 0.44–0.60; *P* < 0.001) (Fig. 3). However, in the MVA the difference was no longer significant (HR, 0.82; 95% CI 0.64–1.05; *P* = 0.115) (Supplementary Table 1). Factors affecting the MVA were maximum invasive tumor size, N stage, grade, and ER status. DDFI by patients experiencing versus not experiencing a local-recurrence event identified that DDFI was similar at 5 years but the hazards separated at 10 years (HR, 0.80; 95% CI 0.54–1.18; *P* = 0.263 and HR, 0.29; 95% CI 0.14–0.62; *P* = 0.001, respectively) (Supplementary Figure 2A). When assessing DDFI by BCS patients with surgical margins of 0 mm versus >0 mm, those with margins >0 mm had a significantly better DDFI compared with those with 0 mm margins (Supplementary Figure 2B). Similar results were also found in OS. UVA of OS by surgical type demonstrated that mastectomy patients had a significantly worse OS compared with BCS (HR, 0.53; 95% CI 0.45–0.62; *P* < 0.001) (Fig. 4), and in the MVA the difference was no longer significant (HR, 0.79; 95% CI 0.61–1.03; *P* = 0.081) (Supplementary Table 2). Excluding ER status, the same factors also affected the MVA for OS. Moreover, when looking at OS by local-recurrence event and by surgical margins, the findings matched those of the DDFI analyses (Supplementary Figure 3A and 3B).

## DISCUSSION

Previous findings from the POSH study reported on the effects of ethnicity and obesity, both of which have affected outcome including DDFI in this young age group,^27,28^ and family history, which has not.^17^ This study has investigated the effect of surgery on LRI, DDFI, and OS, and the effect of local recurrence on DDFI and OS in young women with breast cancer.

A large number of studies use inconsistent definitions of local-recurrence, often not specifying which events have been included in the local-recurrence definition.^29^ This study has therefore clearly described the definition of local recurrence in the methods with criteria outlining which events were included/excluded depending on the time of competing events. This study also incorporated the use of FPSMs to assess the time-varying effect of surgical type on LRI.

Previous findings from a meta-analysis of RCTs conducted by the EBCTCG^18^ presented first recurrence rates (local and distant) which appeared much higher after BCS in younger women; 36.1% for women <40 years undergoing BCS with radiotherapy. However, the number analyzed was relatively small (n = 363) and there was no breakdown in the meta-analysis of local versus distant recurrences. In this study, the majority of events were found to be distant and not local (139 LRI vs 752 DDFI events) which demonstrates the predominant risk in these young patients is of distant and not local recurrence.

Although UVA demonstrated worse DDFI and OS for mastectomy compared with BCS, this is almost certainly because of imbalances in prognostic features between the groups. Patients undergoing mastectomy had significantly larger tumors than BCS, with a higher proportion of tumors EIC+, and ER+, PR+, and/or HER2+. Unsurprisingly, tumors of maximum invasive tumor size >30 mm, N1 stage, and grade 3 were significant factors in both DDFI and OS MVA, the differences between surgical type for DDFI and OS were no longer significant after correction for these factors. Interestingly, maximum overall (invasive + in situ) tumor size >30 mm was not a significant factor in either OS or DDFI MVA models, indicating that whereas overall tumor size influences surgical decision making, invasive tumor size is the relevant size parameter predicting DDFI and OS. A sensitivity analysis excluding patients with a maximum overall tumor size >30 mm showed similar results in a UVA of LRI comparing surgical type.

The results of this study support existing literature in regards to both OS and DDFI by surgical type, with no evidence of surgical type affecting survival or distant-disease in this age group. This is consistent with RCTs comparing mastectomy versus BCS in the breast cancer population as a whole^4–6,11^ indicating that surgical choices for younger women can use accepted criteria without impacting outcome.

The results of this study also demonstrated similar LRR in the first 18 months for mastectomy and BCS, but a larger disparity is seen at 5 and 10 years, with significantly higher LRR for BCS. The clinical implication is that, at least initially, for local recurrence there is no disadvantage in treating young women surgically with BCS in general and no evidence that BCS leads to a disadvantage in DDFI or OS. It is not yet possible to comment beyond 10 years at this stage for this cohort. However, other studies suggest LRR continue to rise beyond 10 years after BCS.^19,30^

An interesting finding from this study is the effect of margin on outcomes. Although no effect was seen for LRI, differences were observed for both OS and DDFI; positive margins were associated with significantly worse OS and DDFI. This could be because of reduced power of LRI outcome because of a fewer local-recurrence events and missing data for margin status, or possibly because of patients with a positive margin being more likely to present with a distant relapse, or combination of distant disease and local recurrence, which would, as defined here, be considered a distant event, as these would not be surgically salvageable, isolated local recurrences. In this analysis, a positive margin was defined according to ASCO guidance as tumor at the margin.^24^ Although 24.5% of margin status information was missing, sensitivity analyses of MVA models using multiple imputation were carried out and showed very similar results to the complete-case analyses. The finding that surgical margins are a factor in the development of distant disease would support the concept of the importance of surgical quality with attention to margins, with re-excision where appropriate. Taken together with the lack of evidence here that oncological surgical type influences distant-relapse it could be argued that completeness of excision is more important than the extent of surgery.

In regards to radiotherapy, patients treated with BCS who were not documented to have adjuvant radiotherapy unsurprisingly had higher LRR, implying that the data were correct (rather than data missing because of patients having radiotherapy elsewhere), and highlighting the importance of radiotherapy as part of breast conservation. Although no effect of radiotherapy boost was shown for patients undergoing BCS, it must be noted that this is not an RCT. Interestingly, provision for radiotherapy boost was not shown to be statistically correlated with margin status; however, the clinical implication we would draw is that, at least in this study, provision of a radiotherapy boost appears to be less important than attention to detail to surgical margins in terms of its effect on LRI and DDFI.

More than 60% of patients undergoing mastectomy received CWR-XRT and a clear association of benefit of CWR-XRT on LRI has been demonstrated here, despite potential confounding. Given results of the most recent Oxford overview^31^ it is likely that thresholds for CWR-XRT after mastectomy are likely to fall further. Given that the majority of young patients are likely to receive radiotherapy, even if surgically treated with mastectomy, there are likely to be implications for reconstructive decision making.

These findings also support the message that avoiding local recurrence is important as increased local recurrence is associated with poorer DDFI and OS.^30,32,33^ Our data suggest that valid strategies to reduce local recurrence might include avoidance of a positive margin after BCS and provision of CWR-XRT after mastectomy where indicated, but do not support mastectomy over BCS where both options are available.

In this analysis, the frequency of local recurrence is much lower than that of distant relapse indicating that the main hazard experienced by these patients, at least within the first 10 years, is of distant rather than local recurrence. We have not demonstrated an impact of tumor stage or biological type on local recurrence in this analysis, possibly because of reduced power because of a lower LRR. In addition to young age, factors recognized to influence local recurrence after BCS and mastectomy include axillary nodal status, margin status, and lack of systemic therapy.^34^ When considering molecular subtype a greater proportion of young women appear to have luminal B tumors^2^; however, young age remains predictive of LRR independent of molecular subtype,^35^ although there is a suggestion that molecular subtype may affect local recurrence.^36^ Younger patients with breast cancer are also more likely to carry a germline BRCA-mutation and it is currently unknown whether this influences LRI or DDFI, although clearly it does increase second new primary breast tumors; and contralateral new primary events were not included in this analysis of local recurrence. Once final genotyping in this cohort has been completed, further analyses will also be performed by BRCA status to see if this has an effect.

Regardless of this, the current Association of Breast Surgery guidelines state that the target local-recurrence rate after surgery should be <3% and not >5% at 5 years.^37^ This study has demonstrated that LRI in younger patients treated by mastectomy would fulfill this criterion (HR, 2.63; 95% CI 1.85–3.74), and that the lower 95% LRI limit for younger women undergoing BCS is within this range (HR, 5.33; 95% CI 4.15–6.85).^37^ Furthermore, our findings are consistent with recommendations for breast surgery within recent consensus guidelines for the management of young women with breast cancer.^38^

A limitation of this study is that as this was not an RCT, any differences/lack of differences in LRI, OS, and/or DDFI were because of the surgical type alone could be the result of confounding. However, we have accounted as far as possible for biases and this is a large prospective cohort representative of cancer treatment in this age group in the United Kingdom.^23^ It should be noted this analysis was performed according to a prespecified plan and LRI was clearly defined to address the inconsistency of reporting in a number of previous studies.^29^

In conclusion, there are no survival advantages for surgical type after adjusting for known prognostic factors. There is no difference in LRI between BCS and mastectomy in young women with breast cancer in the short-term but, beyond 18 months, LRR are higher after BCS. Local recurrence is associated with increased risk of distant relapse, and in patients undergoing BCS, a positive surgical margin increases the likelihood of a distant relapse. For those undergoing mastectomy, CWR-XRT reduces the likelihood of distant relapse. Surgical extent therefore appears less important for DDFI than completeness of excision or, where appropriate, CWR-XRT. Future work will assess the impact of germline genotype on LRI, distant relapse, and OS.

## Supplementary Material

Supplemental Digital Content
