# Supplementary material for: Local Recurrence and Breast Oncological Surgery in Young Women With Breast Cancer: The POSH Observational Cohort Study
Source: Ann Surg. 2016 Jul 27;266(1):165–72. doi: 10.1097/SLA.0000000000001930 (PMC5639998; doi:10.1097/SLA.0000000000001930)
Supplement: Supplemental Digital Content [file ansu-266-165-s001.doc]

i
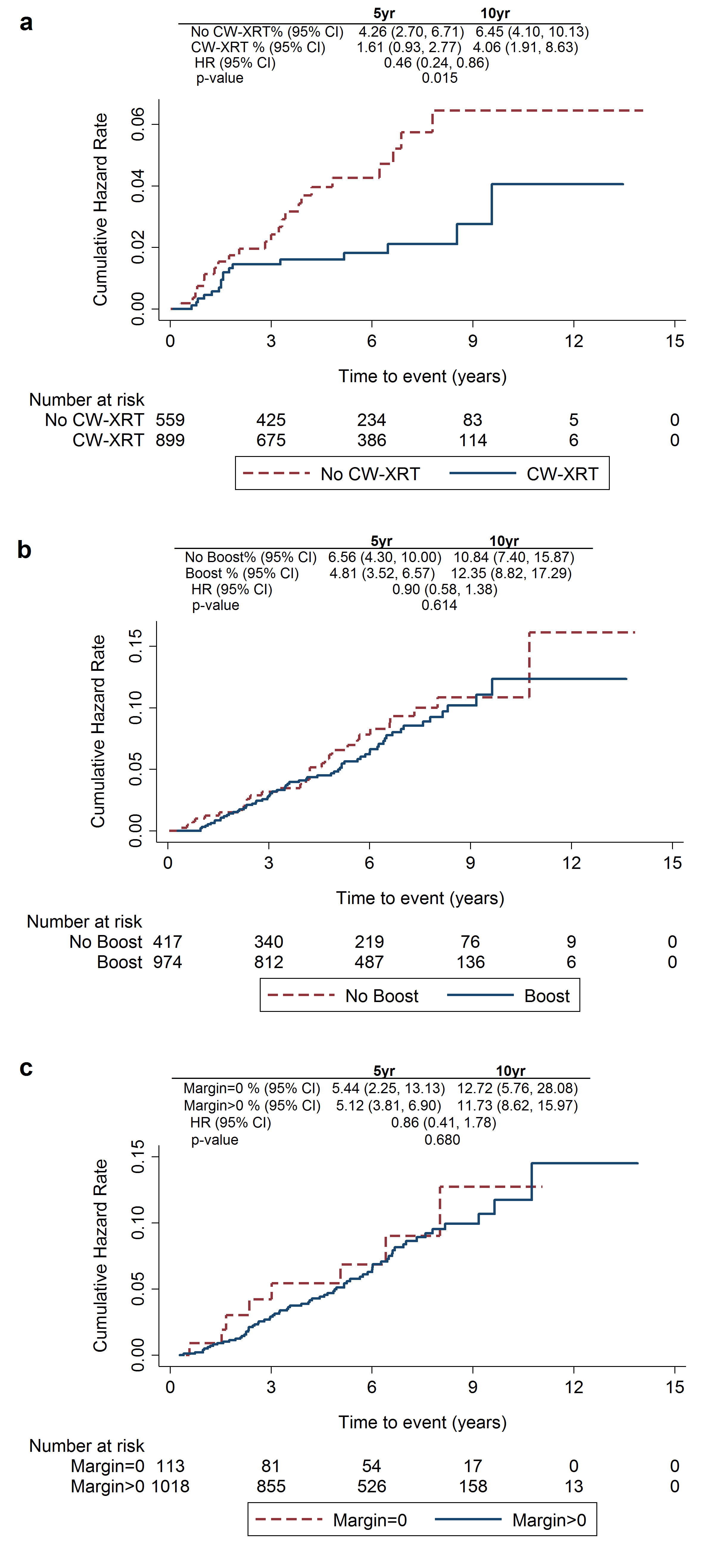


**SUPPLEMENTARY FIGURE 1.** Local-recurrence interval Nelson-Aalen cumulative hazard plot for: a all patients undergoing mastectomy by chest-wall radiotherapy; b all patients undergoing breast conserving surgery by radiotherapy boost; and c all patients undergoing breast conserving surgery by surgical margin.


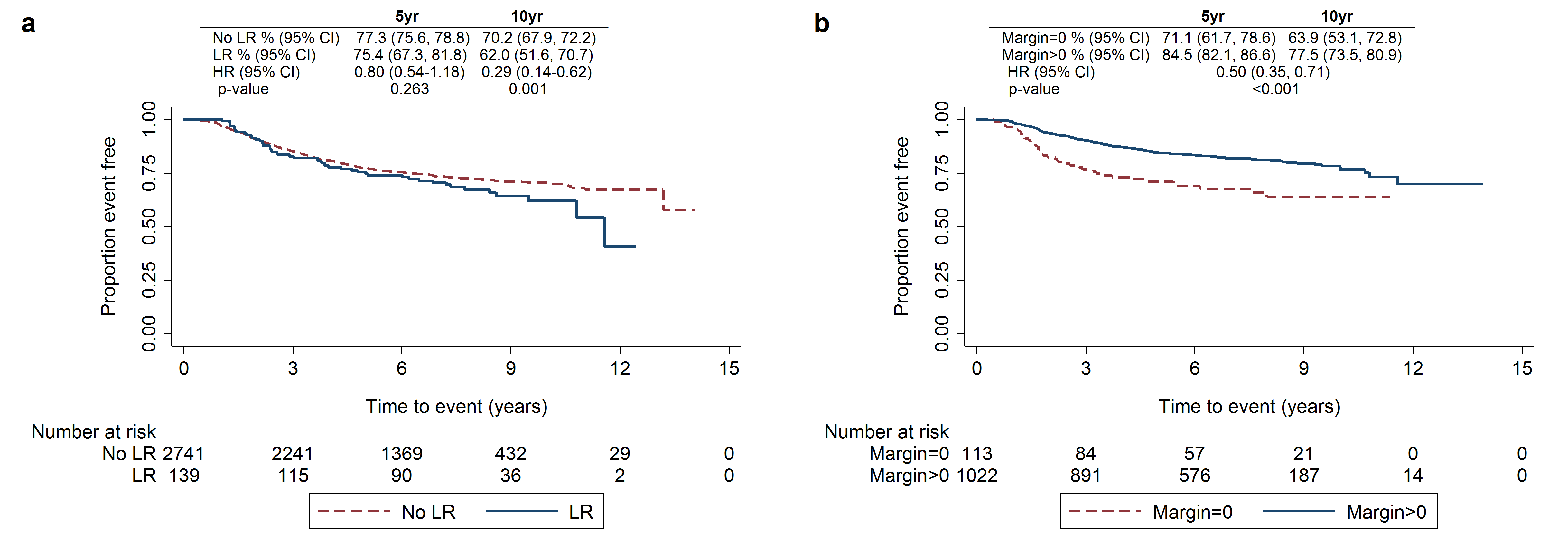


**SUPPLEMENTARY FIGURE 2.** Distant disease free interval Kaplan-Meier plot for: a all patients by Local-recurrence event; and b all patients undergoing breast conserving surgery by surgical margin.


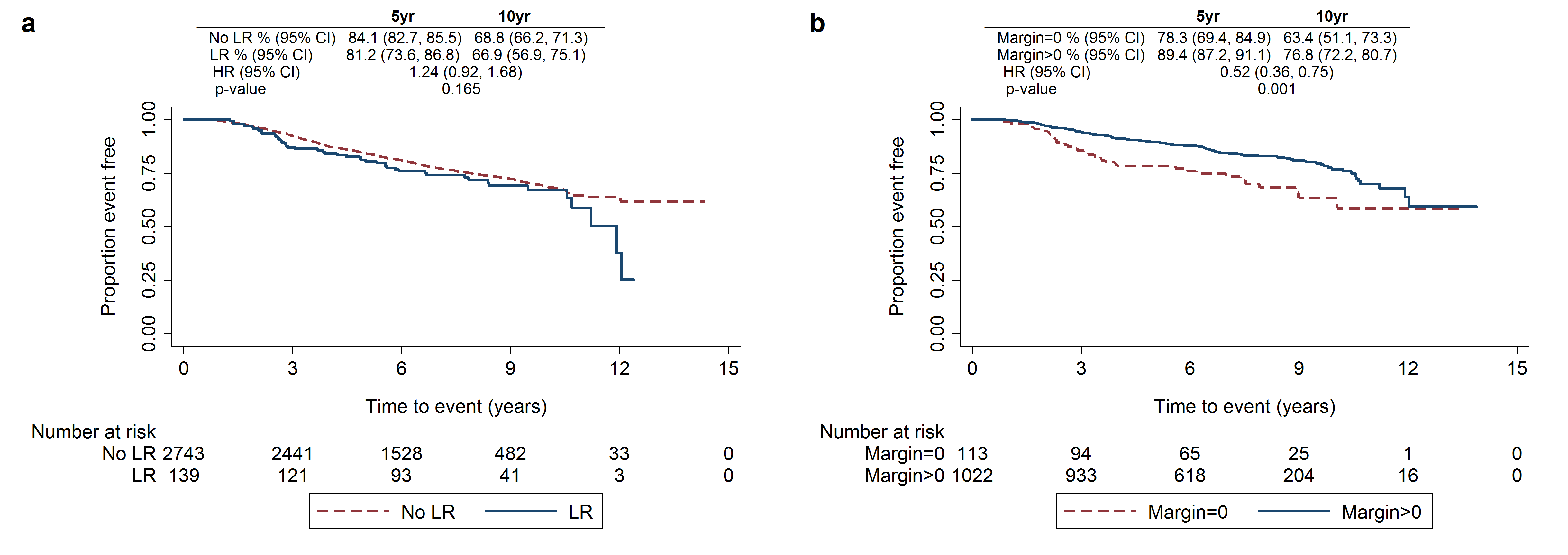


**SUPPLEMENTARY FIGURE 3.** Overall survival Kaplan-Meier plot for: a all patients by Local-recurrence event; and b all patients undergoing breast conserving surgery by surgical margin.

| **SUPPLEMENTARY TABLE 1.** Distant disease free interval Cox proportional hazards model multivariable analysis results for all patients (excluding those with neoadjuvant chemotherapy), stratified by adjuvant hormone therapy and surgical margin | | | |
| --- | --- | --- | --- |
| **Covariate** | **HR†** | **95% CI** | **p-value** |
| **Surgical type** |  |  |  |
| Mastectomy | 1 (Ref. cat.) | - | - |
| BCS (unadjusted) | 0.51 | 0.44 to 0.60 | <0.001 |
| BCS (adjusted) | 0.82 | 0.64 to 1.05 | 0.115 |
| **Age at diagnosis, in years (continuous)** | 0.98 | 0.95 to 1.01 | 0.139 |
| **Maximum overall (invasive+*in situ*) tumour size** |  |  |  |
| ≤30mm | 1 (Ref. cat.) | - | - |
| >30mm | 0.96 | 0.66 to 1.39 | 0.811 |
| **Maximum invasive tumour size** |  |  |  |
| ≤30mm | 1 (Ref. cat.) | - | - |
| >30mm | 1.79 | 1.25 to 2.57 | 0.001 |
| **Focality** |  |  |  |
| Localised | 1 (Ref. cat.) | - | - |
| Multifocal | 0.99 | 0.79 to 1.26 | 0.963 |
| **N stage** |  |  |  |
| N0 | 1 (Ref. cat.) | - | - |
| N1 | 2.33 | 1.85 to 2.95 | <0.001 |
| **Histological Grade** |  |  |  |
| 1 | 1 (Ref. cat.) | - | - |
| 2 | 2.07 | 0.96 to 4.45 | 0.064 |
| 3 | 2.69 | 1.25 to 5.76 | 0.011 |
| **ER Status** |  |  |  |
| Negative | 1 (Ref. cat.) | - | - |
| Positive | 1.42 | 1.01 to 2.00 | 0.042 |
| **HER2 Status** |  |  |  |
| Negative | 1 (Ref. cat.) | - | - |
| Positive | 1.19 | 0.96 to 1.48 | 0.114 |
| **Adjuvant radiotherapy** |  |  |  |
| No/unknown | 1 (Ref. cat.) | - | - |
| Yes | 0.86 | 0.63 to 1.17 | 0.335 |
| HR=Hazard Ratio, CI=Confidence Interval, ER=Oestrogen Receptor, HER2=Human Epidermal growth factor Receptor 2.  † Unless otherwise stated, HR presented for the multivariable (adjusted) model. | | | |

| **SUPPLEMENTARY TABLE 2.** Overall survival Cox proportional hazards model multivariable analysis results for all patients (excluding those with neoadjuvant chemotherapy), stratified by adjuvant hormone therapy and surgical margin | | | |
| --- | --- | --- | --- |
| **Covariate** | **HR†** | **95% CI** | **p-value** |
| **Surgical type** |  |  |  |
| Mastectomy | 1 (Ref. cat.) | - | - |
| BCS (unadjusted) | 0.53 | 0.45 to 0.62 | <0.001 |
| BCS (adjusted) | 0.79 | 0.61 to 1.03 | 0.081 |
| **Age at diagnosis, in years (continuous)** | 0.98 | 0.95 to 1.01 | 0.147 |
| **Maximum overall (invasive+*in situ*) tumour size** |  |  |  |
| ≤30mm | 1 (Ref. cat.) | - | - |
| >30mm | 0.94 | 0.63 to 1.39 | 0.740 |
| **Maximum invasive tumour size** |  |  |  |
| ≤30mm | 1 (Ref. cat.) | - | - |
| >30mm | 1.66 | 1.14 to 2.43 | 0.009 |
| **Focality** |  |  |  |
| Localised | 1 (Ref. cat.) | - | - |
| Multifocal | 0.97 | 0.75 to 1.24 | 0.783 |
| **N stage** |  |  |  |
| N0 | 1 (Ref. cat.) | - | - |
| N1 | 2.45 | 1.91 to 3.15 | <0.001 |
| **Histological Grade** |  |  |  |
| 1 | 1 (Ref. cat.) | - | - |
| 2 | 2.88 | 1.05 to 7.86 | 0.039 |
| 3 | 4.17 | 1.54 to 11.34 | 0.005 |
| **ER Status** |  |  |  |
| Negative | 1 (Ref. cat.) | - | - |
| Positive | 1.37 | 0.96 to 1.95 | 0.085 |
| **HER2 Status** |  |  |  |
| Negative | 1 (Ref. cat.) | - | - |
| Positive | 1.10 | 0.88 to 1.38 | 0.405 |
| **Adjuvant radiotherapy** |  |  |  |
| No/unknown | 1 (Ref. cat.) | - | - |
| Yes | 1.04 | 0.74 to 1.45 | 0.839 |
| HR=Hazard Ratio, CI=Confidence Interval, ER=Oestrogen Receptor, HER2=Human Epidermal growth factor Receptor 2.  † Unless otherwise stated, HR presented for the multivariable (adjusted) model. | | | |
